# Second Primary Spindle Cell Carcinoma of the Tongue: A Rare Histology

**DOI:** 10.7759/cureus.27175

**Published:** 2022-07-23

**Authors:** Lalchhandami Colney, Chinmayee Panigrahi, Mahesh Sultania, Amit Kumar Adhya

**Affiliations:** 1 Department of Surgical Oncology, All India Institute of Medical Sciences, Bhubaneswar, Bhubaneswar, IND; 2 Department of Pathology and Laboratory Medicine, All India Institute of Medical Sciences, Bhubaneswar, Bhubaneswar, IND

**Keywords:** head and neck squamous cell cancer, spindle cell mesenchymal tumor, tongue neoplasm, second primary malignancy, oral cancer pathology

## Abstract

Spindle cell carcinoma (SpCC) is a rare variant of poorly differentiated squamous cell carcinoma (SCC), characterised by the presence of both squamous (carcinomatous) and spindle cell (sarcomatous) elements. Early detection and improvement in treatment for oral SCC lead to prolonged survival, thereby increasing the frequency of second primary tumours (SPTs) in the oral cavity. In this paper, we report a case of SpCC of the tongue in a 62-year-old male with a history of SCC; the right lateral border of his tongue status post-treatment completion four years ago, now presented with a polypoidal growth over the tip of his tongue for four months. An immunohistochemical study revealed features suggestive of SpCC (spindle cell pattern of cells, expression of vimentin, immunopositivity for cytokeratin (membranous), and focally positive for p40 (nuclear)). To the best of our knowledge, this is the first reported case of a spindle cell variant of SCC presenting as a second primary in an oral cancer survivor patient.

## Introduction

The increased survival and meticulous follow-up after treatment of patients with oral squamous cell carcinoma (SCC) have led to an increased frequency of second primary tumours (SPT). Spindle cell carcinoma (SpCC) is one of the rare variants of oral SCC. It is an aggressive tumour with frequent local recurrence and metastasis [1]. This is the first case of the spindle cell variant of SCC as a second primary in an oral cancer patient.

## Case presentation

**Figure 1:**
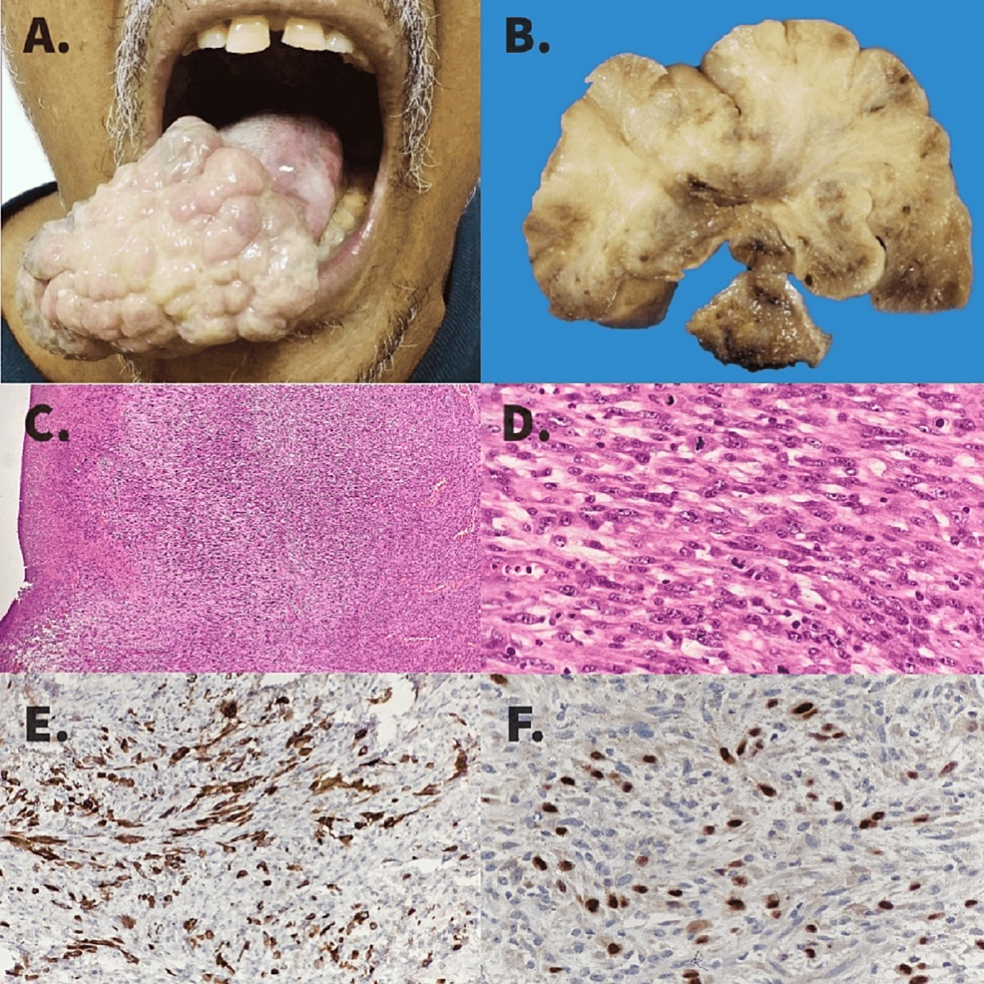
Second Primary Spindle Cell Carcinoma Tongue (A) Polypoidal growth at the tip of the tongue. (B) Gross photograph of the tumor, showing a polypoidal fleshy growth with a stalk attached to the tip of the tongue. The surface is ulcerated, and the cut surface is soft, grayish-white with focal hemorrhages. (C) Photomicrograph showing surface ulceration and a spindle cell tumor; H & E stain, 40×. (D) Photomicrograph showing spindle cell tumor, the tumor cells show moderate nuclear pleomorphism, vesicular chromatin, and brisk mitosis; H & E stain, 400×. (E) The tumor cells are immunopositive for pancytokeratin, indicating its epithelial differentiation, 400×. (F) Tumor cells are immunopositive for p40, indicating its squamous differentiation, tumor cells were negative for S-100, SMA, Desmin, CD34, and Bcl2, 400×.

A 62-year-old man, a tobacco chewer, presented with a polypoidal growth at the tip of the tongue for four months (history of carcinoma on the right lateral border of the tongue, four years back; underwent wide local excision with primary closure of the defect and right supra-omohyoid neck dissection; final histopathological evaluation: well-differentiated SCC, T2N0M0, margins negative, no adjuvant therapy). On examination, a 5 cm × 5 cm polypoidal growth with a 1 cm stalk was noted at the tip of the tongue, more than 2 cm away from the previous surgery scar, and no cervical nodes were palpable (Figure 1A). The punch biopsy was suggestive of a poorly differentiated malignant tumor. He underwent wide local excision of the tongue lesion with primary closure of the defect and left supra-omohyoid neck dissection. The intraoperative frozen section of the margins was negative for malignancy. His postoperative period was uneventful and he was discharged in stable condition on postoperative day 5.

Histopathological examination revealed a 6.5 cm × 4.0 cm × 3.0 cm polypoidal growth with a stalk. The external surface was lobulated with cleft-like spaces. The cut section showed a solid grayish-white surface with a variegated appearance with focal areas of haemorrhage with no necrosis (Figure 1B). The distance between the tumour and the stalk margin was 8 mm, and the stalk margin was free of tumor. Microscopic examination showed extensive areas of surface ulceration with granulation tissue along with sub epithelium showing oval to elongated moderately pleomorphic cells arranged in a herringbone pattern and short and long fascicles. Cells showed scanty cytoplasm, coarsely clumped chromatin, and one to two prominent nucleoli. The tumour cells were immunopositive for CK (membranous) and focally positive for p40 (nuclear) and negative for EMA, CD34, BCL2, CK, and SMA (Figure 1C-1F). Fifteen nodes were identified in the neck dissection specimen, all free of tumor. The final histopathological diagnosis was spindle cell variant SCC with stage T3N0M0. He was advised to have adjuvant radiation therapy and is currently on follow-up after treatment completion.

## Discussion

SpCC is rare and seen in 1-3% of SCCs of the head and neck. These tumours are biphasic, having both carcinomatous and sarcomatous components. Previously, the two cell types were considered to arise from separate stem cells colliding to form a single tumour (collision tumor); another theory considered the sarcomatoid spindle cell component as an atypical reactive stromal proliferation; hence, these tumours were also called pseudosarcoma. However, recently, the monoclonal theory has been accepted. Both the components originate from a single stem cell, and the de-differentiation or metaplastic alteration of the epithelial component leads to spindle cell formation [2]. Males in their sixth and seventh decades with a history of alcohol or tobacco consumption (both chewed and smoked) are predominantly affected. A history of prior radiation exposure to the head and neck is also considered a predisposing factor. The buccal mucosa is the most common site in the oral cavity, and involvement of the tongue is infrequently reported [3].

On review of the literature, no case of spindle cell carcinoma as a recurrence in an oral cancer survivor patient was identified. Hence, we searched for patients with spindle cell carcinoma in the oral cavity, and a total of 189 cases of oral SpCC were reported, with 47 cases being tongue primary (Table 1).

**Table 1:** Spindle Cell Carcinoma of Oral Cavity Reported in Literature NA: not applicable

Reference	No. of cases	
	Oral cavity	Subsite - tongue
Romanach et al. [1]	5	1
Someren et al. [2]	1	1
Viswanathan et al. [3]	65	13
Tataka et al. [4]	4	2
Leventon and Evans [5]	8	2
Su et al. [6]	15	5
Ellis and Corio [7]	59	12
Lane [8]	4	2
Gelfman and Williams [9]	1	1
Kessler Bartley [10]	1	1
Slootweg et al. [11]	2	2
Chen et al. [12]	1	1
Reyes et al. [13]	2	2
Biradar et al. [14]	1	1
Rosko et al. [15]	19	NA
Silva et al. [16]	1	1

SpCC is associated with a polypoid exophytic growth pattern, rapid growth with or without pain, ulceration, or bleeding, as observed in our case. Histologically, the sarcomatoid spindle cell components form the majority of the tumour bulk, and the carcinomatous component may be focal or inconspicuous [4]. Immunohistochemistry usually showed vimentin positivity in the spindle cells due to the loss of keratin. IHC for keratin and keratin subunits should also be done for the epithelial components since not all SpCC may be positive with keratin stain. The spindle cell component might resemble other benign and malignant head and neck diseases such as chronic ulceration with granulation tissue, radiation-induced atypia, and mucosal polyps with stromal atypia. Hence, proper evaluation with IHC is mandatory. In the present case, the mesenchymal marker vimentin is expressed, and the tumour cells are immunopositive for cytokeratin (membranous) and focally positive for p40(nuclear); however, CD34, S100, SMA, and Desmin are negative.

SpCC has been reported to have a higher local recurrence, shorter disease-specific survival, and lower overall survival than SCC of the oral cavity and oropharynx. A tumour's size and depth of invasion, presence of regional metastases, and previous history of radiotherapy are considered a poor prognosis, with superficial or early-stage tumours having better overall survival than deeply invasive tumours [5,6]. Surgical resection with neck dissection is accepted as the best treatment of choice in the oral cavity and aims to control local and regional recurrence [7]. Due to the high local recurrence rate, Su et al. have proposed excision with a more comprehensive (2 cm) margin [6]. Surgical intervention, with or without radiotherapy, has a better prognosis than radiotherapy alone. Locally advanced tumours have a higher chance of distant metastasis, the most common site being the lung. The role of adjuvant chemotherapy is mostly reserved for distant metastasis; its role in reducing the risk of recurrence or metastasis is still debatable.

Oral cancer survivors are significantly more prone to developing a second primary cancer than the general population. The theories of "field cancerization" and "multicentric origin of epidermoid carcinoma" are widely accepted as an explanation for the occurrence of second primary tumours [17]. Min et al. [18] reported a significantly increased risk of second primary cancers in oral cavity cancer survivors, stressing typical site distribution and the benefit of a surveillance protocol. Survivors with a continuous tobacco and alcohol consumption history are more prone to developing second primary tumors. Patients who receive only radiation or surgery for primary cancer are more likely to continue their addictions, as observed in the present case. The above factors play a role in forming second primary tumours in oral cancer patients.

## Conclusions

The improvement in diagnosis and multimodality treatment approach has led to improved survival in patients with oral squamous cell carcinoma. However, due to field cancerization, second-primary tumors remain the leading cause of long-term mortality. Patients with oral cavity cancer were found to have SPT commonly located in the head and neck. Our case report mentioned the occurrence of SPT in the tongue with an index oral cavity cancer (tongue). However, the occurrence of the spindle cell variant of squamous cell carcinoma as a second primary tumor has not been reported to our knowledge. The diagnosis of SpCC is a histopathological challenge and is crucial for proper management. Oral cancer survivors should be diligently followed up for a long time with proper tobacco and alcohol de-addiction treatment and a low index of suspicion for recurrence or second primary tumour in high-risk individuals.
